# Exploring the Efficacy of the Occupational Therapy-Fundamental Motor Skills Training (OT-FMST) Protocol on Functional Performance in Children With Autism Spectrum Disorder

**DOI:** 10.7759/cureus.94310

**Published:** 2025-10-10

**Authors:** Jolly G Jain, R.K. Sharma, Shivani Bhardwaj, Saba Irem, Amit Dwivedi

**Affiliations:** 1 Occupational Therapy, Santosh Deemed to be University, Ghaziabad, IND; 2 Occupational Therapy, Jamia Hamdard University, New Delhi, IND; 3 Orthopedics, Santosh Deemed to be University, Ghaziabad, IND

**Keywords:** autism spectrum disorder, functional performance, fundamental motor skills, motor development, occupational therapy, sensory integration

## Abstract

Background

Autism spectrum disorder (ASD) is primarily characterized by deficits in social communication and restricted behaviors, but motor impairments are also highly prevalent and debilitating. Children with ASD often experience challenges in fundamental motor skills (FMS), particularly in coordination, postural control, and locomotor abilities. These impairments not only limit physical activity but also restrict social interaction, play, and communication, thereby compounding developmental challenges. Although sensory integration and FMS-based interventions have each shown positive outcomes, few studies have systematically combined them.

Methods

This study was conducted at the Santosh Occupational Therapy Department and included 60 children aged 3-7 years diagnosed with ASD (Indian Scale for Assessment of Autism (ISAA) scores 70-153). Participants were conveniently assigned to control and experimental groups. Both groups received occupational therapy (OT) focused on sensory integration, while the experimental group additionally underwent structured FMS training. Sessions were conducted four times per week for 12 weeks, lasting 45-50 minutes for the control group and 60-65 minutes for the experimental group. Primary outcome measures included the ISAA and the Pediatric Evaluation of Disability Inventory (PEDI), which assessed functional skills and caregiver assistance in self-care, mobility, and social domains.

Results

After the intervention, the functional performance of the OT-FMST group improved more than that of the control group. PEDI scores showed significant improvements in self-care skills across all levels (p < 0.05). Social functional skills also improved significantly across all score metrics (p < 0.01). Mobility skills improved significantly in raw and normative scores (p < 0.05), while scaled scores showed a positive but nonsignificant trend. Caregiver assistance needs were notably reduced in the experimental group, with significant improvements in self-care (raw and normative), mobility (scaled and normative), and all social assistance measures (p < 0.01). These findings suggest that the addition of FMS training promoted independence and functional gains beyond those achieved with sensory integration therapy alone.

Conclusions

The OT-FMST protocol was effective in enhancing functional skills and reducing caregiver dependence in children with ASD. Integrating structured FMS training with conventional OT provides a more comprehensive intervention approach, supporting improvements in self-care, mobility, and social participation. These results highlight the clinical value of incorporating FMS-based training into OT programs for children with ASD.

## Introduction

The International Classification of Diseases, 11th Revision (ICD-11), and the Diagnostic and Statistical Manual of Mental Disorders, Fifth Edition (DSM-5), both describe autism spectrum disorder (ASD) as a pervasive neurodevelopmental condition characterized by impairments in social interaction and communication, along with the presence of repetitive and restricted behaviors that make it difficult for individuals to communicate and socialize with others [1,2].

Both recent and earlier studies highlight that movement problems are a significant component of ASD [3,4]. Although motor impairments are not included in the current diagnostic criteria, they are increasingly recognized as prevalent and impactful, particularly in children exhibiting nonverbal deficits and repetitive behaviors [4]. These difficulties often emerge early, worsen with age, and restrict functional and social development.

Neuroimaging studies have shown that children with ASD exhibit reduced volume in the central segment of the corpus callosum (CC), a region essential for interhemispheric communication. This reduction has been linked to increased repetitive behaviors and poorer motor coordination skills, suggesting that altered interhemispheric connectivity via the CC may underlie both core autistic features and associated motor deficits [3]. Recent research further indicates that motor abnormalities are not merely co-occurring features but are intricately linked to the core symptoms of ASD [5].

Children with ASD acquire fundamental motor skills (FMS) significantly more slowly than neurotypical children. Evidence shows that these delays not only stand out but also influence multiple aspects of development. Delayed motor skills are associated with lower physical activity levels and can negatively affect communication, language acquisition, and social interaction abilities [6]. Among the various motor-related challenges seen in ASD, difficulties in FMS, encompassing both gross and fine motor abilities, are particularly pronounced [7]. These challenges often manifest as deficits in motor coordination, poor postural control, and delayed motor development, frequently linked to atypical maturation of the prefrontal cortex.

FMS are essential for developing movement competence, facilitating play, and promoting lifelong engagement in physical activity. Key FMS include walking, running, jumping, catching, and throwing. Impairments in these foundational skills not only hinder motor proficiency but also affect broader developmental areas such as cooperation, joint attention, empathy, emotional regulation, and social interaction. Moreover, the social difficulties common in ASD can further limit opportunities for physical activity, underscoring a bidirectional relationship between motor and social development [6-8].

Earlier research has shown that the movement skills of children with ASD are more impaired than expected for their cognitive level, suggesting that these deficits reflect not just delays but underlying impairments [9]. Various intervention strategies have been developed to address motor deficits in ASD. While many programs include elements of sensory integration, brain gym exercises, aerobic activities, educational games, and sports, they often address FMS only indirectly [10].

A pilot study investigating the feasibility of FMS intervention compared two groups of children with ASD and examined the efficacy of a parent-mediated approach for improving and maintaining FMS. The results indicated that parents can play a significant role in enhancing FMS in children with ASD, highlighting the value of home-based or parent-led motor therapies [11]. A systematic review and meta-analysis concluded that exercise interventions significantly improve FMS in children with ASD, with large effects for locomotor skills and moderate effects for object control and stability skills [8].

Occupational therapy (OT) is widely recognized as a key intervention for children with ASD, emphasizing the importance of equitable and effective services across health and education settings. A qualitative study involving Canadian occupational therapists reported the use of diverse interventions targeting restricted and repetitive behaviors in children with ASD, guided by frameworks such as the person-environment-occupation model and applied behavior analysis [12]. Similarly, a study in South Africa found that clinical practices align with global trends, emphasizing informal play-based assessments, sensory processing approaches, and Ayres Sensory Integration (ASI^®^) treatment [13]. Other studies also report that sensory integration interventions significantly reduce emotional and behavioral problems, including hyperactivity, aggression, anxiety, and attention issues, in children with ASD [14].

Although existing literature supports the effectiveness of both sensory integration and FMS interventions in improving functional outcomes in children with ASD, these approaches have largely been studied in isolation [6-14]. To date, no studies have systematically examined their combined effects, positioning the present study as a novel contribution to the field. The current study, therefore, investigates the effectiveness of combining OT with FMS training (OT-FMST) in children with ASD. Integrating OT with the FMST protocol was designed to address deficits in children with ASD and enhance functional skills. We hypothesized that combining FMS with OT would lead to greater improvements in functional skills and self-care performance compared to OT alone.

## Materials and methods

Research design

This study was conducted at the Santosh Occupational Therapy Department. The study procedures were approved by the university’s Institutional Research Advisory Board. A test-retest design was employed to compare the effects of conventional OT with those of an integrated intervention combining OT and the OT-FMST protocol in children with ASD. The study was conducted from September 1, 2024, to March 15, 2025.

Participants

A total of sixty children (N = 60), aged 3 to 7 years, were enrolled through convenience sampling. The Indian Scale for Assessment of Autism (ISAA) was used to confirm ASD, with eligible participants scoring between 70 and 153. Children with additional physical or intellectual disabilities, congenital anomalies, metabolic disorders, or other comorbid conditions were excluded.

Following enrollment, participants were conveniently allocated into two groups, experimental (n = 30) and control (n = 30). Inclusion criteria included a confirmed ASD diagnosis by a qualified healthcare professional, normal or corrected vision and hearing, intact airways, and no history of seizures. Children scoring between 70 and 153 on the ISAA were included. To prevent therapy overlap, children receiving concurrent interventions were excluded.

Written informed consent was obtained from parents or legal guardians prior to participation. Children demonstrating poor compliance with therapy sessions were withdrawn based on predefined criteria. To ensure consistency, intervention procedures were standardized across therapists through structured training sessions, manuals, and session-specific checklists. A senior occupational therapist monitored treatment fidelity using a fidelity checklist to assess randomly selected sessions.

Based on previous research suggesting that focused, high-repetition motor exercises can enhance engagement and reduce fatigue in children with ASD, each experimental session lasted 15 minutes. The control group, in contrast, received traditional sensory integration therapy (SIT), standard clinical practice, for 45-50 minutes. This deliberate variation in session duration was intended to determine whether a focused, time-efficient approach could yield outcomes comparable to or better than conventional therapy.

Outcome measures

ISAA - Screening Tool

The ISAA is an objective tool used to evaluate and diagnose autism through observation, clinical evaluation, and direct interaction. It includes 40 items assessing various aspects of behavior and development, each scored on a 5-point scale ranging from 1 (never) to 5 (always), with higher scores indicating greater symptom severity. Total scores range from 40 to 200, interpreted as follows: normal (<70), mild autism (70-106), moderate autism (107-153), and severe autism (>153). The ISAA demonstrates excellent psychometric properties, with test-retest reliability between 0.93 and 0.99 and inter-rater reliability of 0.99 [15].

Pediatric Evaluation of Disability Inventory (PEDI) - Outcome Measure

The PEDI assesses functional performance and capability in children with disabilities aged six months to 7.5 years across three domains: (1) self-care, (2) mobility, and (3) social function. It consists of two scales: the Functional Skills (FS) scale, with 197 items, and the Caregiver Assistance and Modifications scale, with 20 items.

The FS scale includes three domains, such as personal care (73 items), mobility (59 items), and social functioning (65 items), scored as 1 (capable) or 0 (incapable). Domain totals are obtained by summing the item scores. The Caregiver Assistance subscale quantifies the level of help required, scored from 0 (completely dependent) to 5 (independent). The Modifications subscale records environmental adaptations or equipment use, categorized as none, child, rehabilitation, or extensive.

The PEDI demonstrates excellent internal consistency, with Cronbach’s alpha coefficients ranging from 0.92 to 0.98 across domains, and high test-retest reliability, with intraclass correlation coefficients between 0.86 and 0.98 [16].

OT intervention

The OT-FMST treatment protocol was implemented over 12 weeks, four times per week. The control group received regular OT based on the child’s individual needs, primarily focused on sensory integration, for 45-50 minutes per session. The experimental group received the same OT along with FMS training for 60-65 minutes per session. Rest breaks were provided as needed (Table 1).

**Table 1:** OT-FMST protocol OT-FMST, Occupational Therapy-Fundamental Motor Skills Training

Serial no.	Duration	Activities	Benefits
1	Phase 1 (Weeks 1-3)	(1) Rocking; (2) rolling; (3) pushing an object; (4) weight transfer; and (5) quadruped back-and-forth	(1) Improves blood circulation, mobilizes muscles, stimulates the balance mechanism of the inner ear, and enhances attentiveness and alertness; (2) enhances balance, develops body awareness, and improves coordination of movements; (3) builds body awareness, especially in the arms; enhances coordination and balancing skills; and boosts spatial and cognitive abilities; (4) develops motor control, promotes bilateral integration, and increases spatial and upper-limb awareness; and (5) strengthens arms, legs, and core muscles through co-contraction, thereby enhancing balance and motor planning skills
2	Phase 2 (Weeks 4-6)	(1) Wrap and rocking; (2) wrap and rolling; (3) obstacle course; (4) pull a dupatta; and (5) squat to stand	(1) Combines the benefits of rocking while calming an overstimulated child and promotes brain development by reducing stress; (2) combines the benefits of rolling while calming an overstimulated child and enhances neural integration and brain development; (3) improves muscle strength, enhances body awareness, develops balance, coordination, and motor planning, and stimulates problem-solving abilities; (4) increases endurance, activates palm alertness, enhances focus, and promotes social skills; and (5) strengthens leg and core muscles, improves cardiovascular endurance, and develops overall body awareness
3	Phase 3 (Weeks 7-9)	1. Crawling; (2) touching opposite toes in the supine lying position; (3) hitting a target while standing on a stool; (4) stepping up and down a stool; and (5) knee walk	(1) Develops balance, enhances body awareness, improves coordination, and stimulates visual tracking; (2) facilitates midline crossing, develops eye-hand-leg coordination, strengthens core muscles, and enhances visual-perceptual skills; (3) improves distance and depth perception, enhances coordination, and develops visual-perceptual skills; (4) promotes depth perception, encourages coordinated reciprocal movements, and supports bilateral integration; and (5) improves joint awareness at the knee, enhances upright posture by activating core strength, and develops coordination and motor planning
4	Phase 4 (Weeks 10-12)	(1) Cat and camel; (2) wall push with hands; (3) wheelbarrow walk; (4) runner’s stretch; and (5) finger touches	(1) Strengthens and stretches core muscles, facilitates alternate flexion and extension of the neck, and enhances visual skill integration; (2) activates palm muscles and receptors, promotes co-contraction of forearm, arm, and scapular muscles, and improves body awareness; (3) strengthens upper-limb muscles, enhances visual integration, and develops motor planning and bilateral integration; (4) stretches lower back and lower-limb muscles, encourages midline crossing, improves eye-hand-leg coordination, and develops overall movement coordination; (5) enhances imitation skills, develops fine motor control, improves finger coordination, and promotes eye-hand integration

Statistical analysis

Data were compiled in a master chart using Microsoft Excel 2021 (Microsoft Corporation, Redmond, WA, USA). All statistical analyses were performed using IBM SPSS Statistics for Windows, Version 20.0 (Released 2011; IBM Corp., Armonk, NY, USA). Descriptive and inferential statistics, including mean, mode, SD, and paired t-tests, were used for analysis. Within-group comparisons were conducted to assess pre- and post-intervention changes, while between-group analyses evaluated differences in treatment effects between the conventional OT and OT + FMST groups. All tests were two-tailed, and statistical significance was set at p < 0.05 unless otherwise specified.

## Results

Demographic data (age comparison between groups)

The analysis of demographic data showed that the mean age score in Group A (control group) (M = 4.75, SD = 1.724) was slightly lower than that in Group B (experimental group) (M = 5.19, SD = 1.114). An unpaired t-test was conducted to examine the difference between the two groups, yielding a t-value of 1.194 with a p-value of 0.2375. As the p-value was greater than the critical value (2.00) at the 0.05 level of significance, the difference in mean age scores between the two groups was found to be statistically insignificant (Table 2).

**Table 2:** Mean age of Group A and Group B This table presents the mean age, SD, and statistical comparison of participants in Group A (control) and Group B (experimental OT-FMST). OT-FMST, Occupational Therapy-Fundamental Motor Skills Training

Unpaired t-test	Comparison	
	Age	
	Group A	Group B
Mean	4.75	5.19
SD	1.724	1.114
Number	30	30
Mean difference	-0.45	
Unpaired t-test	1.194	
p-Value	0.2375	
Table value at 0.05	2	
Result	Not significant	

Self-care functional skills

**Figure 1:**
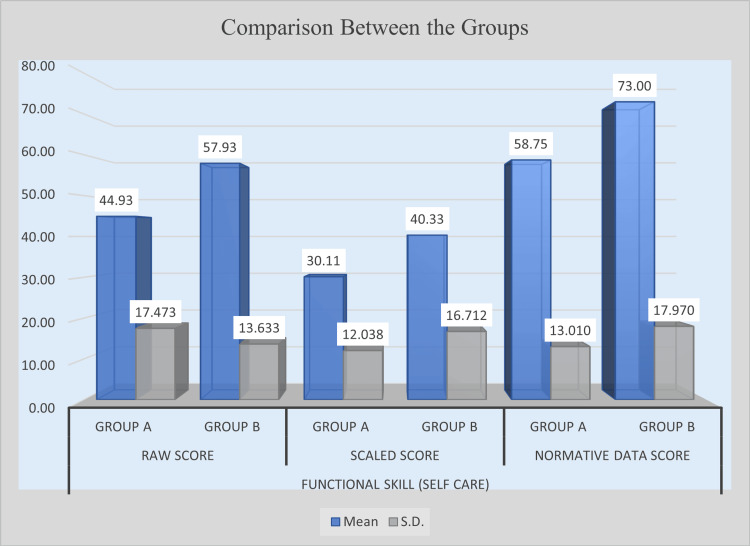
Post-intervention comparison of self-care functional skill scores between experimental and control groups

Following the intervention, the experimental group (Group B) demonstrated significantly greater improvement in self-care functional skills compared with the control group (Group A). In terms of raw scores, Group B obtained a higher mean score (M = 57.93) than Group A (M = 44.93), with a mean difference of 13.00. Statistical analysis revealed a t-value of 3.213 and a p-value of 0.0021, indicating a statistically significant difference. Similarly, for scaled scores, Group B achieved a mean of 40.33, whereas Group A scored 30.11, showing a mean difference of 10.21 with a t-value of 2.527 and a p-value of 0.0150, confirming statistical significance. In normative data scores, Group B recorded a mean of 73.00, while Group A achieved 58.75. The mean difference of 14.25, supported by a t-value of 3.517 and a p-value of 0.0009, indicates a highly significant improvement. These findings suggest that the intervention was highly effective in enhancing self-care functional abilities in children in the experimental group. Table 3 presents the post-intervention comparison of self-care functional skills between Group A and Group B, and Figure 1 illustrates the post-intervention improvement in self-care functional skills.

**Table 3:** Self-care functional skill scores (post-intervention) PEDI self-care scores (mean ± SD) for the control (Group A) and experimental (Group B) groups. Group B showed significant improvements across raw, scaled, and normative scores (p < 0.05). PEDI, Pediatric Evaluation of Disability Inventory

Unpaired t-test	Functional skill (self-care)					
	Raw score		Scaled score		Normative data score	
	Group A	Group B	Group A	Group B	Group A	Group B
Mean	44.93	57.93	30.11	40.33	58.75	73
SD	17.473	13.633	12.038	16.712	13.01	17.97
Number	30	30	30	30	30	30
Maximum	71	73	68.4	66.1	85.1	100
Minimum	11	32	10.8	9.99	33	50.3
Range	60	41	57.6	56.11	52.1	49.7
Mean difference	13		10.21		14.25	
Unpaired t-test	3.213		2.527		3.517	
p-Value	0.0021		0.015		0.0009	
Table value at 0.05	2		2.01		2	
Result	Significant		Significant		Significant	

Mobility functional skills 

**Figure 2:**
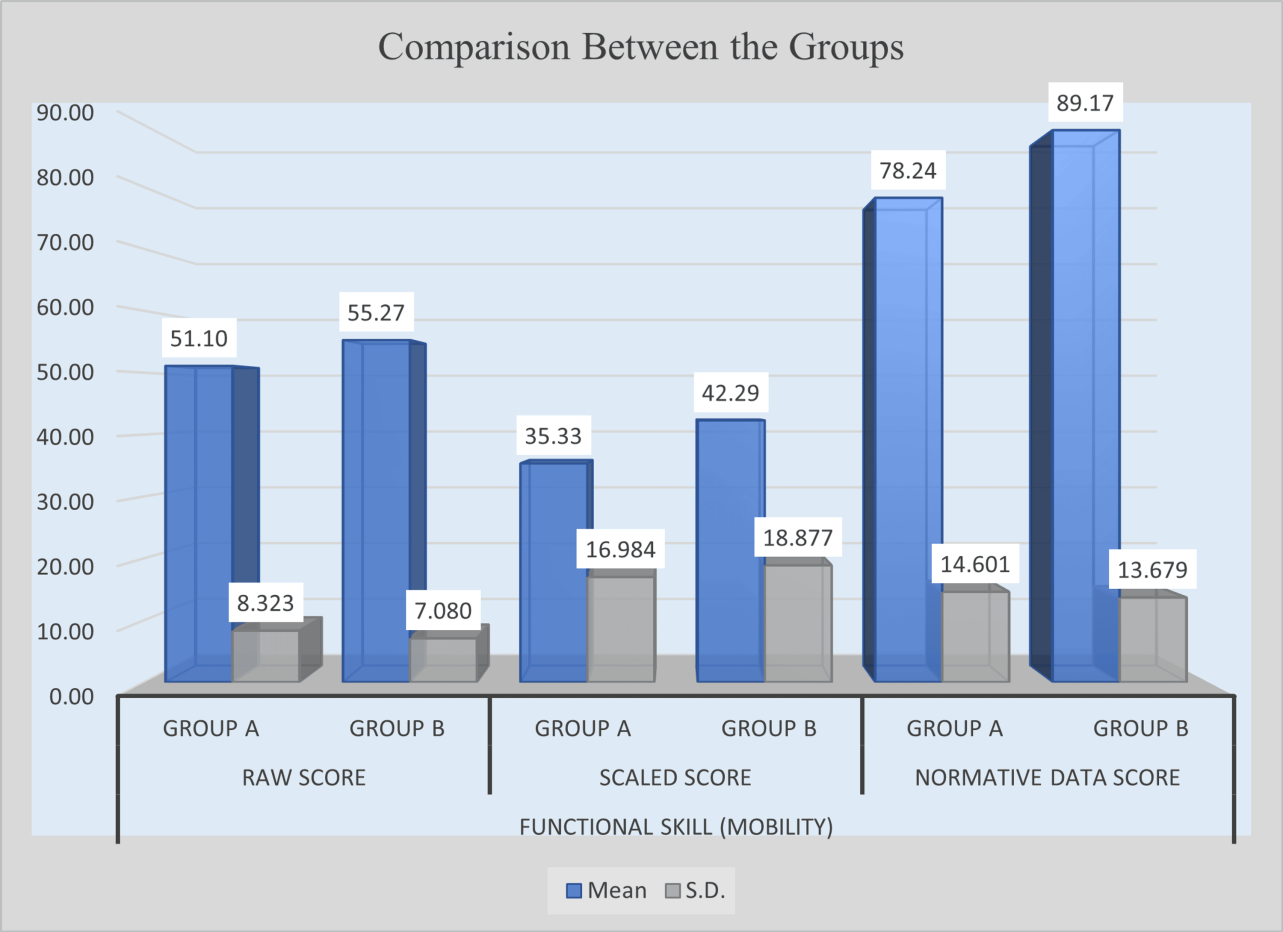
Post-intervention comparison of mobility functional skill scores between experimental and control groups

Post-intervention results revealed noticeable improvements in mobility-related functional skills in the experimental group; however, not all comparisons reached statistical significance. Group B achieved a higher mean raw score (M = 55.27) than Group A (M = 51.10), with a mean difference of 4.17. The corresponding t-value of 2.089 and p-value of 0.0411 indicate that this difference was statistically significant. In contrast, although Group B outperformed Group A in scaled scores (M = 42.29 vs. M = 35.33), the mean difference of 6.95 did not reach statistical significance (t = 1.500, p = 0.1391). However, a significant difference was observed in normative data scores, where Group B obtained a mean of 89.17 compared with Group A’s 78.24. The mean difference of 10.93, supported by a t-value of 2.991 and a p-value of 0.0041, confirms a statistically significant improvement. These results suggest that the intervention positively influenced mobility functional skills, particularly in raw and normative data measures, while scaled scores showed improvements that were not statistically significant. Table 4 presents the post-intervention comparison of mobility functional skills between Group A and Group B, and Figure 2 illustrates the post-intervention improvement in mobility functional skills.

**Table 4:** Mobility functional skill scores (post-intervention) PEDI mobility scores (mean ± SD). Group B showed significant improvements in raw (p = 0.0411) and normative (p = 0.0041) scores; scaled scores were not significant. PEDI, Pediatric Evaluation of Disability Inventory

Unpaired t-test	Functional skill (mobility)					
	Raw score		Scaled score		Normative data score	
	Group A	Group B	Group A	Group B	Group A	Group B
Mean	51.1	55.27	35.33	42.29	78.24	89.17
SD	8.323	7.08	16.984	18.877	14.601	13.679
Number	30	30	30	30	30	30
Maximum	59	59	63.8	69.8	100	100
Minimum	33	26	9.99	9.99	53.9	47.9
Range	26	33	53.81	59.81	46.1	52.1
Mean difference	4.17		6.95		10.93	
Unpaired t-test	2.089		1.5		2.991	
p-Value	0.0411		0.1391		0.0041	
Table value at 0.05	2		2		2	
Result	Significant		Not significant		Significant	

Social functional skills

**Figure 3:**
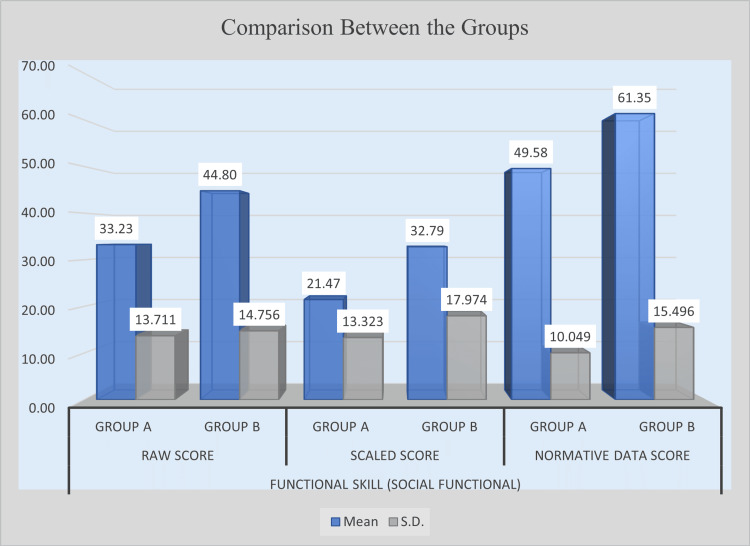
Post-intervention comparison of social function functional skill scores between experimental and control groups

Analysis of social functional skills revealed substantial improvement in the experimental group compared with the control group across all scoring measures. In terms of raw scores, Group B achieved a mean of 44.80, notably higher than Group A’s mean of 33.23. The difference of 11.57 was statistically significant (t = 3.145, p = 0.0026). Similarly, in scaled scores, Group B recorded a mean of 32.79, while Group A scored 21.47. The mean difference of 11.32 was also statistically significant (t = 2.772, p = 0.0075). A significant improvement was evident in normative data scores, where Group B’s mean score was 61.35 compared with 49.58 for Group A. The mean difference of 11.77, supported by a t-value of 3.492 and a p-value of 0.0009, demonstrates a highly significant enhancement. These findings confirm that the intervention effectively improved social functional skills in children within the experimental group. Table 5 presents the post-intervention comparison of social functional skills between Group A and Group B, and Figure 3 illustrates the post-intervention improvement in social functional skills.

**Table 5:** Social function functional skill scores (post-intervention) PEDI social function scores (mean ± SD). Group B showed significant improvements across all score types (p < 0.01). PEDI, Pediatric Evaluation of Disability Inventory

Unpaired t-test	Functional skill (social function)					
	Raw score		Scaled score		Normative data score	
	Group A	Group B	Group A	Group B	Group A	Group B
Mean	33.23	44.8	21.47	32.79	49.58	61.35
SD	13.711	14.756	13.323	17.974	10.049	15.496
Number	30	30	30	30	30	30
Maximum	59	65	56.8	82.3	70.8	100
Minimum	12	14	9.99	9.99	26	37.9
Range	47	51	46.81	72.31	44.8	62.1
Mean difference	11.57		11.32		11.77	
Unpaired t-test	3.145		2.772		3.492	
p-Value	0.0026		0.0075		0.0009	
Table value at 0.05	2		2		2	
Result	Significant		Significant		Significant	

Caregiver assistance for self-care

**Figure 4:**
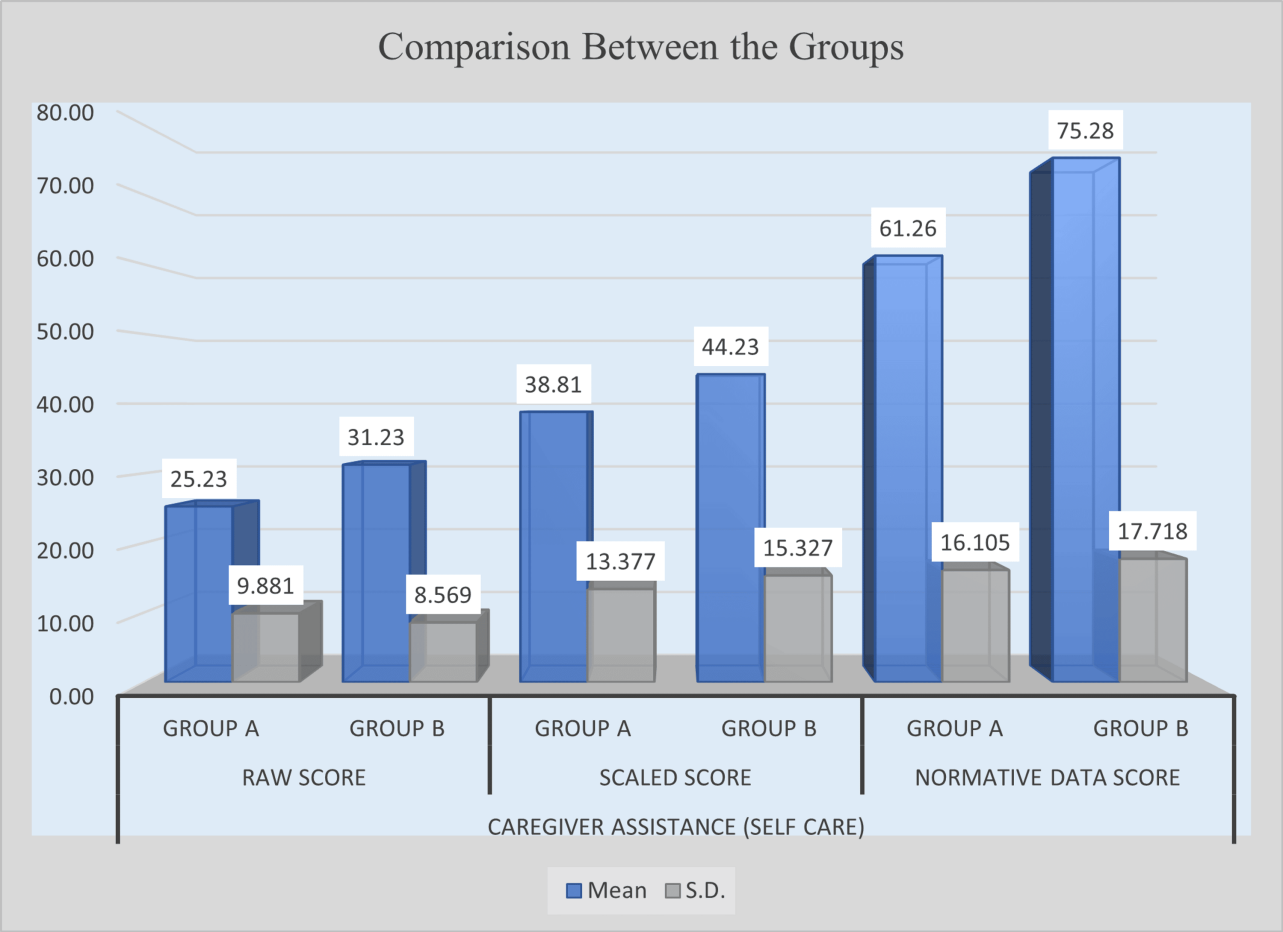
Post-intervention comparison of caregiver assistance in self-care between experimental and control groups

The results for caregiver assistance in self-care demonstrated that the experimental group benefited significantly from the intervention. In raw scores, Group B achieved a mean of 31.23 compared with Group A’s 25.23, yielding a mean difference of 6.00. This difference was statistically significant (t = 2.513, p = 0.0148). In scaled scores, Group B scored higher (M = 44.23) than Group A (M = 38.81), but the mean difference of 5.42 did not reach statistical significance (t = 1.459, p = 0.1499). However, in normative data scores, Group B recorded a mean of 75.28, substantially higher than Group A’s 61.26. The difference of 14.03, supported by a t-value of 3.209 and a p-value of 0.0022, indicates a significant improvement. Overall, the intervention positively impacted caregiver assistance in self-care, with significant gains observed in raw and normative data scores. Table 6 presents the post-intervention comparison of caregiver assistance for self-care between Group A and Group B, and Figure 4 illustrates the post-intervention improvement in caregiver assistance for self-care.

**Table 6:** Caregiver assistance in self-care (post-intervention) Caregiver assistance scores in self-care. Group B improved significantly in raw (p = 0.0148) and normative (p = 0.0022) scores; scaled scores were not significant.

Unpaired t-test	Caregiver assistance (self-care)					
	Raw score		Scaled score		Normative data score	
	Group A	Group B	Group A	Group B	Group A	Group B
Mean	25.23	31.23	38.81	44.23	61.26	75.28
SD	9.881	8.569	13.377	15.327	16.105	17.718
Number	30	30	30	30	30	30
Maximum	38	40	68.2	64.6	83.2	100
Minimum	0	13	9.99	9.99	0	47.3
Range	38	27	58.21	54.61	83.2	52.7
Mean difference	6		5.42		14.03	
Unpaired t-test	2.513		1.459		3.209	
p-Value	0.0148		0.1499		0.0022	
Table value at 0.05	2		2		2	
Result	Significant		Not significant		Significant	

Caregiver assistance for mobility

**Figure 5:**
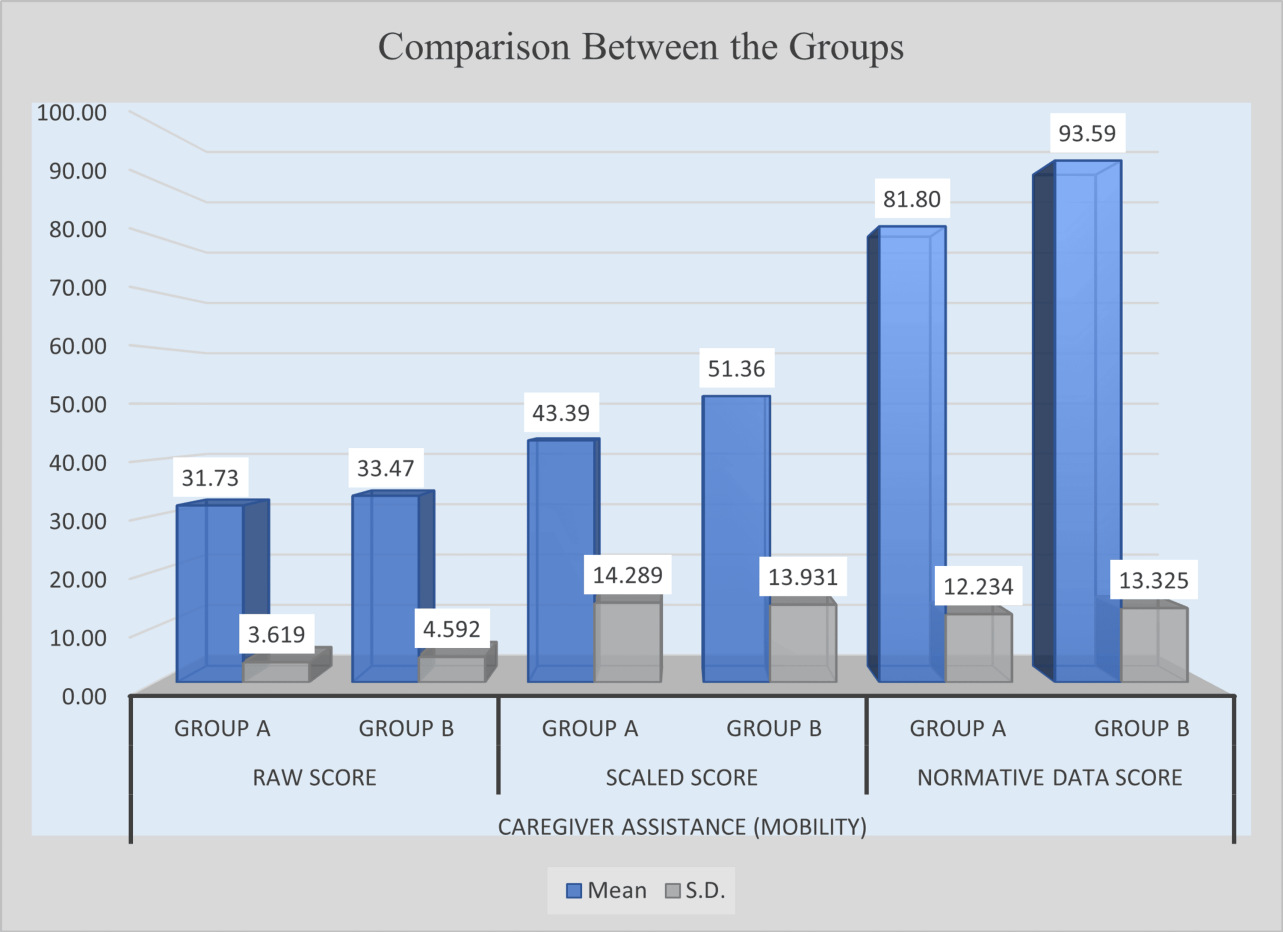
Post-intervention comparison of caregiver assistance in mobility between experimental and control groups

The analysis of caregiver assistance in mobility revealed mixed outcomes. Although Group B obtained a slightly higher raw score (M = 33.47) than Group A (M = 31.73), the mean difference of 1.73 was not statistically significant (t = 1.624, p = 0.1098). Conversely, in scaled scores, Group B scored significantly higher (M = 51.36) compared with Group A (M = 43.39). The mean difference of 7.97, supported by a t-value of 2.188 and a p-value of 0.0327, indicates a significant improvement. A similar trend was observed in normative data scores, where Group B achieved a mean of 93.59, surpassing Group A’s 81.80. The mean difference of 11.80, confirmed by a t-value of 3.572 and a p-value of 0.0007, reflects a highly significant enhancement. These results suggest that the intervention was effective in improving caregiver assistance for mobility, particularly in scaled and normative measures. Table 7 presents the post-intervention comparison of caregiver assistance for mobility between Group A and Group B, and Figure 5 illustrates the post-intervention improvement in caregiver assistance for mobility.

**Table 7:** Caregiver assistance in social function (post-intervention) Caregiver assistance social function scores. Group B showed significant improvements across all score types (p < 0.05).

Unpaired t-test	Caregiver assistance (social function)					
	Raw score		Scaled score		Normative data score	
	Group A	Group B	Group A	Group B	Group A	Group B
Mean	14.23	18.9	36.48	44.86	59.27	74.01
SD	6.442	5.268	14.222	13.561	18.348	16.628
Number	30	30	30	30	30	30
Maximum	25	25	73.6	66.3	100	100
Minimum	1	7	9.99	17.4	11.3	42.9
Range	24	18	63.61	48.9	88.7	57.1
Mean difference	4.67		8.38		14.73	
Unpaired t-test	3.072		2.336		3.259	
p-Value	0.0032		0.023		0.0019	
Table value at 0.05	2		2		2	
Result	Significant		Significant		Significant	

Caregiver assistance for social functional skills

**Figure 6:**
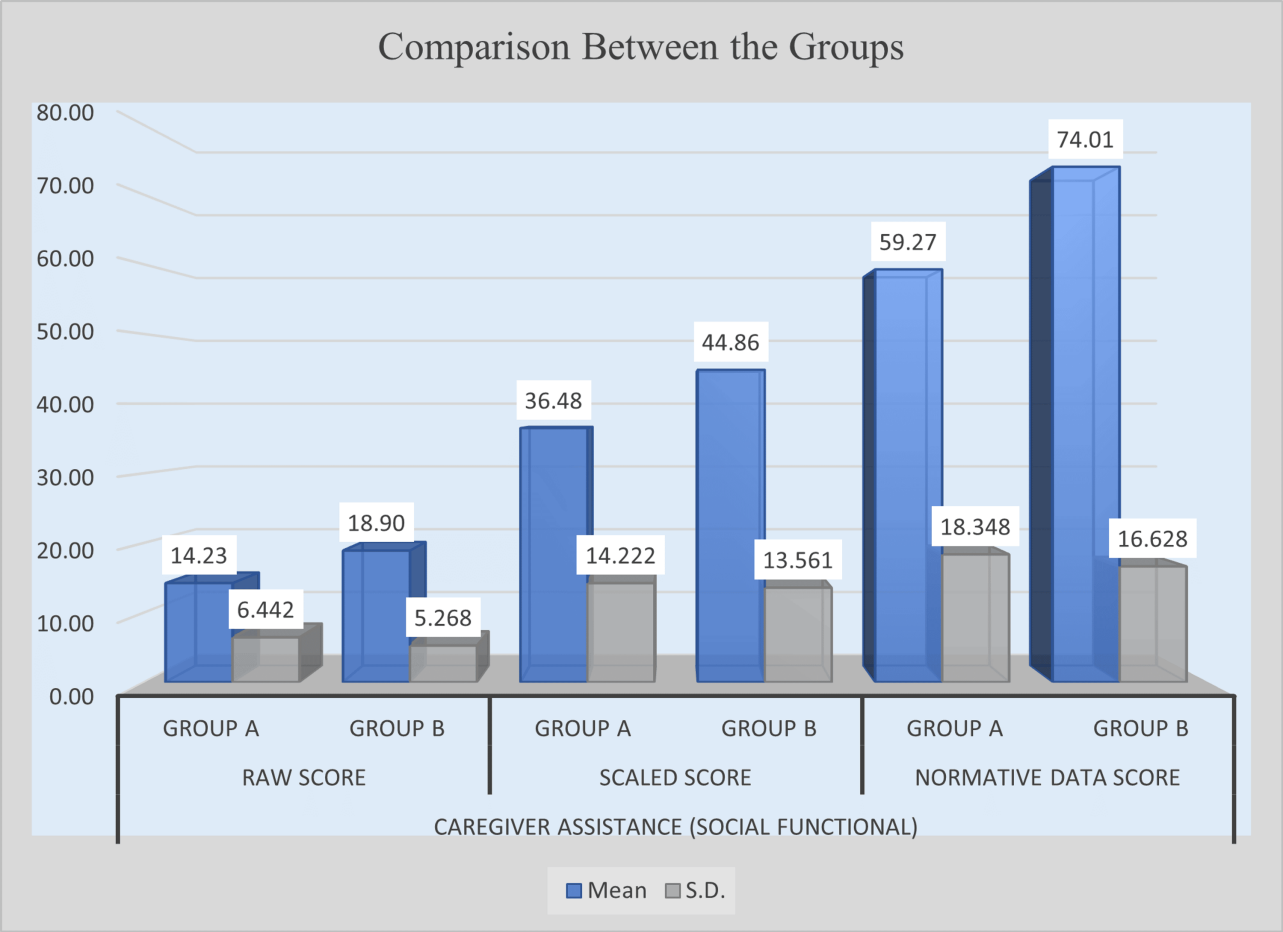
Post-intervention comparison of caregiver assistance in social function between experimental and control groups

Post-intervention results indicated significant gains in caregiver assistance for social functional skills in the experimental group. In raw scores, Group B obtained a mean of 18.90, higher than Group A’s 14.23, and the mean difference of 4.67 was statistically significant (t = 3.072, p = 0.0032). Similarly, in scaled scores, Group B scored 44.86, outperforming Group A (36.48). The mean difference of 8.38 was also statistically significant (t = 2.336, p = 0.0230). In normative data scores, Group B achieved a mean of 74.01 compared with Group A’s 59.27, resulting in a mean difference of 14.73, which was highly significant (t = 3.259, p = 0.0019). These findings indicate that the intervention successfully enhanced caregiver assistance in social functional skills, showing significant improvements across raw, scaled, and normative data scores. Table 8 presents the post-intervention comparison of caregiver assistance for social functional skills between Group A and Group B, and Figure 6 illustrates the post-intervention improvement in caregiver assistance for social functional skills.

**Table 8:** Caregiver assistance in mobility (post-intervention) Caregiver assistance mobility scores. Group B improved significantly in scaled (p = 0.0327) and normative (p = 0.0007) scores; raw scores were not significant.

Unpaired t-test	Caregiver assistance (mobility)					
	Raw score		Scaled score		Normative data score	
	Group A	Group B	Group A	Group B	Group A	Group B
Mean	31.73	33.47	43.39	51.36	81.8	93.59
SD	3.619	4.592	14.289	13.931	12.234	13.325
Number	30	30	30	30	30	30
Maximum	35	35	70.5	70.5	100	100
Minimum	17	11	9.99	9.99	52.3	44.3
Range	18	24	60.51	60.51	47.7	55.7
Mean difference	1.73		7.97		11.8	
Unpaired t-test	1.624		2.188		3.572	
p-Value	0.1098		0.0327		0.0007	
Table value at 0.05	2		2		2	
Result	Not significant		Significant		Significant	

## Discussion

This pre- and post-experimental study was conducted on children with ASD, with outcomes assessed using the PEDI scale before and after the intervention. A total of 60 children were recruited and divided into control and experimental groups. Thirty children received only OT intervention, while the experimental group received OT-FMST. The intervention lasted for 12 weeks, with sessions conducted four times per week.

Previous literature has emphasized the prevalence of sensory processing difficulties and dysfunctions in children with ASD [13,16]. SIT, based on Ayres’ 1972 theory (ASI^®^), is a commonly used OT approach aimed at improving a child’s ability to process and integrate sensory information, thereby promoting more organized and adaptive behaviors [17-19]. One study reported a prevalence of sensory processing disorder ranging from 65% to 95% [18].

Similar findings were reflected in our study, as the control group, which received SIT as part of OT, showed improvements in all PEDI subsets: self-care, mobility, and social function. However, the experimental group that received OT combined with FMS training demonstrated more significant gains than the control group. To date, there are no previous studies that have specifically combined FMS and OT interventions. The present results, therefore, provide novel evidence that integrating these two approaches yields greater improvements in functional skills, as measured by pre- and post-intervention PEDI scores.

In this study, significant improvements were observed across all domains, except in the mobility scaled scores under functional skills, caregiver assistance in self-care scaled scores, and caregiver assistance in mobility raw scores.

Furthermore, several studies have highlighted that children with ASD exhibit more pronounced delays in acquiring FMS compared to typically developing children. The findings from this study align with those reports, suggesting that the performance of FMS among children with ASD remains considerably delayed by late childhood. Children with ASD have been found to demonstrate greater impairments in FMS competencies, particularly in object control and locomotor skills, than both typically developing peers and those with other developmental disorders [20].

Bremer et al. (2015) conducted a pilot study to determine whether an FMS intervention could improve motor skills, adaptive behavior, and social skills in four-year-olds with ASD, and whether different intervention intensities produced varying effects. The results, which parallel our findings, showed that the FMS intervention improved motor skills in the experimental group more than in the control group. All children improved their motor abilities from pre- to post-test and maintained these gains at the six-week follow-up. Similarly, the OT-FMST intervention in our study improved motor, adaptive, and social skills, as well as the children’s capacity for play. These results indicate that FMS interventions can enhance motor abilities in young children with ASD [21].

Another study also reported that exercise interventions may effectively improve FMS in children with ADHD and ASD. The form of the intervention and its timing, frequency, and duration were identified as important moderator variables that positively influenced FMS outcomes. These findings are consistent with the present study, suggesting that OT-FMST interventions may have a beneficial impact on the motor proficiency of children with autism [8,20,21].

Limitations

This study has certain limitations that should be acknowledged. First, the research could be strengthened by adopting a randomized controlled trial (RCT) design. The limited sample size and single-site recruitment restrict the generalizability of the findings. Second, while the chit-based randomization method was practical, it may not provide the same level of rigor as computer-generated randomization. Additionally, variations in parental engagement and the lack of blinding for participants or therapists regarding group assignments could have influenced the outcomes. Future studies should address these factors to enhance methodological robustness.

## Conclusions

This study provides preliminary evidence that integrating FMS training with conventional OT significantly enhances functional performance in children with ASD. Compared to OT alone, the OT-FMST protocol led to greater improvements in self-care, mobility, and social domains, as well as reduced caregiver dependence. These findings highlight the importance of addressing motor deficits alongside sensory processing difficulties in ASD rehabilitation. The study underscores the clinical utility of combining sensory integration approaches with structured FMS training to promote independence, participation, and overall quality of life in young children with ASD. Future research involving larger samples, RCTs, and long-term follow-up is recommended to further validate and generalize these findings.
